# Noise and opinion dynamics: how ambiguity promotes pro-majority consensus in the presence of confirmation bias

**DOI:** 10.1098/rsos.231071

**Published:** 2024-04-24

**Authors:** Peter Steiglechner, Marijn A. Keijzer, Paul E. Smaldino, Deyshawn Moser, Agostino Merico

**Affiliations:** ^1^ Leibniz Centre for Tropical Marine Research (ZMT), Bremen, Germany; ^2^ Constructor University, Bremen, Germany; ^3^ Institute for Advanced Study in Toulouse, Toulouse School of Economics, Toulouse, France; ^4^ Department of Cognitive and Information Sciences, University of California Merced, Merced, CA, USA; ^5^ Santa Fe Institute, Santa Fe, NM, USA

**Keywords:** opinion formation, agent-based model, noise, bounded confidence, climate change, computational social science

## Abstract

Opinion dynamics are affected by cognitive biases and noise. While mathematical models have focused extensively on biases, we still know surprisingly little about how noise shapes opinion patterns. Here, we use an agent-based opinion dynamics model to investigate the interplay between confirmation bias—represented as bounded confidence—and different types of noise. After analysing where noise can enter social interaction, we propose a type of noise that has not been discussed so far, ambiguity noise. While previously considered types of noise acted on agents either before, after or independent of social interaction, ambiguity noise acts on communicated messages, assuming that socially transmitted opinions are inherently noisy. We find that noise can induce agreement when confirmation bias is moderate, but different types of noise require quite different conditions for this effect to occur. An application of our model to the climate change debate shows that at just the right mix of confirmation bias and ambiguity noise, opinions tend to converge to high levels of climate change concern. This result is not observed with the other types. Our findings highlight the importance of considering and distinguishing between the various types of noise and the unique role of ambiguity in opinion formation.

## 1. Introduction

Social influence plays a critical role in how people form opinions [1–3], including important topics like climate change, public health measures (like wearing face masks to prevent the spread of COVID-19) or government-funded social measures. However, social influence entails more than high-fidelity transmission of information; it is affected by cognitive biases and by noise. The term ‘cognitive biases’ refers to systematic deviations from classically rational use of information^1^ . For example, confirmation bias is a well-documented phenomenon where people disproportionately select the information that confirms their prior beliefs [5]. The role of biases in opinion formation has been studied extensively and is typically associated with impeding the convergence of opinions (although, under certain conditions, biases may have the opposite effect, e.g. [6,7]). Noise represents unsystematic, random factors in the opinion formation processes. Although well studied [8–10], the role of noise in social influence remains less obvious than the role of biases and has triggered controversies (e.g. the recent exchange between Kahnemann *et al*. and Krakauer *et al*. [11]).

To study what drives the emergence of certain opinion patterns in society, mathematical modelling and, in particular, agent-based modelling have become vital tools (refer to [12–14] for recent reviews of this field). Models of social influence typically account for cognitive biases by assuming, for instance, that agents perceive information through a biased filter [15], that agents make systematic errors when they adapt to social influence [16,17] or that agents ignore dissonant opinions [18,19]. By including biases in the social influence process, the models generate virtual societies in which either disagreement persists or consensus can form [12].

Noise is a source of stochasticity in an otherwise deterministic opinion-formation process. If models do not take noise into account, they run the risk of misrepresenting real phenomena on both individual and collective levels [10]. For example, many models assume that agents always apply the same, predefined heuristics during social interactions. In reality, when interacting with others, people apply a diverse set of social learning strategies depending on the context [3]. Models without noise also assume that agents have accurate representations of their own or others’ opinions, but in reality, people often construct an opinion on the spot [20,21], making social interaction inherently noisy. Moreover, most models that ignore noise produce patterns in which both individual opinions and macro-patterns cease to change when the virtual society has reached an equilibrium. In reality, individual opinions remain dynamic, and although public opinion distributions are often quite stable [22], we do observe drifting or fission–fusion dynamics in real life, for example, in the climate change debate [23]. Such macro-dynamics contradict the convergence towards a stable equilibrium obtained in computational models without noise.

Noise can capture a variety of real-world processes, which can all be formalized in different ways. Here, we provide an overview of how different formalizations of noise affect the dynamics of opinion formation. We survey the literature and present a taxonomy of four types of noise underlying the social influence process: (i) selectivity noise, (ii) adaptation noise, (iii) exogenous noise, and (iv) ambiguity noise. The first three have been the object of study in previous models of opinion formation (see §3 for a brief review of the literature on each type of noise), but have not been subject to rigorous comparison. We present this comparison, conceptually and numerically, using computational experiments. Previous studies considered noise as acting either on the connection between two agents by varying who tolerates whom as interaction partner (selectivity noise), on the receiver of a message by changing how the opinion of the receiver changes after its influence-response (adaptation noise) or on any agent, in general, by occasionally perturbing its opinion regardless of social interaction (exogenous noise). The fourth and novel type of noise—ambiguity noise—acts on the communicated message, reflecting the idea that socially transmitted information is often ambiguous by nature. This idea relates to prominent work in psychology [20,21,24], economics [25], communication sciences [26,27] or sociology [28]. The concept of ambiguity noise has appeared previously, for example, in a model on interpersonal relations [29], but its effects on consensus formation in the presence of biased assimilation have not been studied.

Ambiguity noise is a general feature of the social influence process, and we argue that this type of noise is particularly relevant in debates on topics about complex matters where there is a ground truth or objectively superior opinion, such as in the debate on the anthropogenic origin of climate change. Social influence is a critical factor in this debate as people signal their opinions to others—often unconsciously—for example, by openly indicating support for public figures on social media or by making everyday decisions, such as dietary choices, which are visible to others [30,31]. When people express opinions in such ways, the signals they send are inherently ambiguous and uncertain and thus prone to create misunderstandings and interpretation errors, especially when dealing with a complex topic like climate change [32]. For example, empirical studies showed that when people were asked about their perception of climate change, the weather or the season at the time of the interview affected their answers and thus concealed their true opinion on climate change to some extent [33,34]. It is not obvious how ambiguity noise affects opinion patterns in such debates. In fact, as we show later, it can both promote or impede consensus depending on the level of noise. Our study, therefore, aims to clarify the role played by ambiguity noise in opinion dynamics models, a largely overlooked aspect.

Several modelling studies have investigated the impact of noise on opinion dynamics and some of them also considered multiple types of noise (e.g. [35,36]). A common feature of these studies is that many prominent findings in social influence models are not robust to the inclusion of noise. For example, the emergence of opinion clustering in societies with biased agents does not occur under even very small levels of noise [9,10,37–39], despite being a common result in models without noise. However, there has been little effort to systematically and comprehensively compare different types of noise and their effects on the opinion patterns emerging from noisy social influence. Such system comparisons are useful; for example, a recent study compared four types of uncertainty on the evolution of social learning and found systematic differences in how each type influenced evolutionary dynamics [40]. Similarly, a study by Grauwin & Jensen [36] investigated two types of noise—exogenous and selectivity noise—finding that they lead to opposite dynamics and interact in non-trivial ways. Although the authors included these two different types of noise, the focus of their work was not to systematically study the interplay of bias and different noises. Our study aims to provide such a systematic comparison of the four different types of noise and to identify their effects on collective opinion patterns in social influence models.

In this study, we present an agent-based model of opinion formation in which the agents are affected by bias and noise. We focus on confirmation bias as one of the most important cognitive biases affecting opinion formation (especially in the debate on climate change [41,42]). We extend the popular model by Deffuant *et al*. [19] to represent this bias as bounded confidence (BC). We present a taxonomy of types of noise, provide implementations in the framework of the BC model and study the dynamics that these types of noise produce for different degrees of bias. We show that noise in this model generally bolsters agreement when the agents are moderately biased. But different types of noise induce quite different patterns of agreement and disagreement. Finally, we apply the model to the debate on climate change by calibrating agents’ opinions to survey data [43]. As a majority of the agents hold pro-environmental attitudes at the outset, one would intuitively expect that unambiguous communication and a high degree of openness to other opinions promote pro-environmental consensus. Surprisingly, we find that a mix of ambiguity noise and confirmation bias provides the best conditions to foster convergence on pro-environmental attitudes. This result is unique to ambiguity noise and does not occur with other types of noise.

## 2. Modelling confirmation bias

Confirmation bias reflects the tendency of people to disproportionately attend to information that confirms their prior beliefs. The BC approach [18,19] is arguably the most popular in representing this bias in formal models^2^ [44,45]. Here, we adopt a version of the BC model by Deffuant *et al*. [19] that considers pairwise interactions and asynchronous updating. In this formulation of the BC model, n agents represent human beings who form their opinions through one-to-one interaction. The opinion of an agent i is represented as a scalar value $x_{i}\in [0,1]$ , denoting, for example, the agent’s concern about climate change, ranging from extremely dismissive ( $x_{i}=0$ ) to extremely alarmed ( $x_{i}=1$ ). When the two agents i and j interact, their opinions $x_{i}$ and $x_{j}$ simultaneously align unless the distance between them exceeds the agents’ confidence bounds,


(2.1) $$\begin{array}{l} x_{i}\mapsto \left\{ \begin{array}{l} x_{i}+\mu \cdot (m_{j}-x_{i})\;\text{if }\left|m_{j}-x_{i}\right|\leq \epsilon \;with\;the\;message\;m_{j}=x_{j}\\ x_{i}\;\;\;\;else \end{array}\right.\\ \; \end{array},$$

where μ is the speed at which opinions converge [19] and ϵ is the confidence bound. A large confidence bound represents a low bias (and vice versa). The message, $m_{j}$ , is the perfectly accurate representation of the opinion $x_{j}$ of agent j .

## 3. Modelling noise

### 3.1. Previously studied types of noise

**Figure 1.**
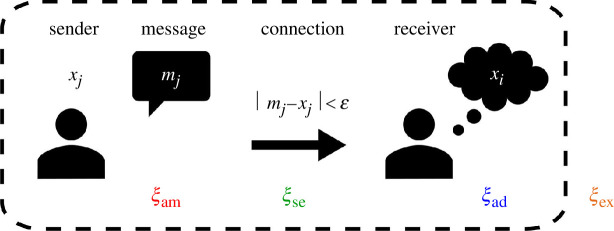
Different types of noise and how they act inside or outside the social interaction process: (i) ambiguity noise, $\xi_{am}$ , acts on the message from a sender, (ii) selectivity noise, $\xi_{se}$ , affects whether a receiver is chosen for interaction in light of the difference between message and receiver opinion and the confidence bound, (iii) adaptation noise, $\xi_{ad}$ , affects the receiver’s opinion after an interaction, and (iv) exogenous noise, $\xi_{ex}$ , perturbs an agent opinion from outside of the social interaction.

In this section, we present the types of noise that have been previously studied: selectivity, adaptation, and exogenous noise. While the precise formalization may differ from model to model, we distinguish these three categories because they describe three distinct phases in the social interaction where noise may enter the system. Figure 1 visually represents where these types of noise enter the system (as well as where ambiguity noise, described in the next section, is involved).

*Selectivity noise* acts on the connection between agents. It stochastically induces interactions between agents that would typically ignore each other or inhibits interactions between agents that would typically influence each other positively (e.g. [9,35,36,38,39,46–48]). Selectivity noise is sometimes seen as a property of the social system and, in analogy to thermodynamics, referred to as ‘social temperature’^3^ [46]. We implemented selectivity noise by adding random (zero-mean Gaussian) fluctuations, $\xi_{se}$ , to the confidence bound, ϵ , of an agent in every interaction with another agent:


(3.1) $$\begin{array}{l} x_{i}\mapsto \left\{ \begin{array}{ll} x_{i}+\mu \cdot (x_{j}-x_{i})\;\text{if }\left|x_{j}-x_{i}\right|\leq \epsilon \;+\xi_{se} & \\ x_{i}\;\;\;\;else \end{array}\right. \end{array}.$$

This reflects the fact that even though confirmation bias will normally select information within a certain confidence bound, individuals are sometimes confronted with and influenced by information outside of their confidence bound. It refers to imperfections or errors in the selection of suitable communication partners.

*Adaptation noise* acts on a receiver by modifying how its opinion changes after a social interaction (e.g. [9,49–52]). We implemented adaptation noise by additionally shifting the agent opinion by a random (zero-mean Gaussian) amount, $\xi_{ad}$ , whenever an agent updates its opinion following a successful interaction with another agent, that is when their prior opinions are within the confidence bound,


(3.2) $$x_{i}\mapsto \left\{ \begin{array}{ll} x_{i}+\mu \cdot (x_{j}-x_{i})+\xi_{ad} & \text{if}\left|\;x_{j}-x_{i}\right|\leq \epsilon \\ x_{i} & else \end{array}\right..$$

Adaptation noise thus describes changes in opinions that are triggered by a change of mind after an interaction and are unrelated to the transmitted message itself.^4^ A socially transmitted message might trigger an individual to autonomously think more about a given topic or may lead to a search for more information. These types of perturbations after a social interaction are captured by adaptation noise.

*Exogenous noise* acts on opinions in a process entirely separate from the social interactions (e.g. [39,53–62]). It may capture an individual’s autonomous development of thought or insights gained from information obtained independently from social interaction. We implement exogenous noise by shifting the agent opinion by a random (zero-mean Gaussian) amount, $\xi_{ex}$ , (an ‘opinion jump’) with some small probability ω ,


(3.3) $$\begin{array}{l} x_{i}\mapsto x_{i}\;+\;\left\{ \begin{array}{ll} \xi_{ex} & with\;probability\;\omega \\ 0 & else \end{array}\right. \end{array}.$$

In all of these implementations, which are conceptually in line with the correspondingly cited model literature, we draw the deviations, $\xi_{ex}$ , $\xi_{se}$ and $\xi_{ad}$ , from a Gaussian distribution N(0,ν) , where ν>0 defines the fixed level of noise. For exogenous noise, we define ω=ν such that the frequency and amplitude of the noisy opinion perturbations are coupled. We ensure that opinions remain within the opinion space, $0\leq x_{i}\leq 1$ , by truncating the Gaussian noise distribution accordingly. In particular, for exogenous and adaptation noise, we resample the noise, ξ , if the posterior opinion $x_{i}$ including this noise would fall outside the opinion space. Note that there are various names for these types of noise in the literature (e.g. what we call ‘adaptation noise’ has been referred to as ‘communication noise’ [52] or ‘individualization’ [9] elsewhere and ‘exogenous noise’ has been referred to as ‘perturbation noise’ [53]). Here, we use names that reflect the different stages of the social interaction process in which noise plays out.

### 3.2. Ambiguity noise

We propose ambiguity in expressed messages as a fourth type of noise. Ambiguity is deeply linked to the conceptualization of opinions. We assume that opinions are fundamentally uncertain estimates of how one should respond in particular scenarios, such as how a person’s concern about climate change may or may not lead to social action depending on how the individual’s uncertainty about the opinion interacts with perceptions of the likely costs and benefits of action. It follows that expressed opinions are likely to be ambiguous signals and, as such, cannot be deterministically quantified on a numeric scale. This can lead to ambiguity on the part of a receiver in terms of how to interpret received information. Another source of ambiguity in socially transmitted messages is that people often construct an opinion on the spot rather than holding predefined opinions [21]. When asked repeatedly, a person may thus produce different opinions on the same topic, a phenomenon called the ‘crowd within’ effect [20,63,64]. This can lead to ambiguity on the part of a sender.

*Ambiguity noise* acts on the message conveying the opinion of the sender. We implement ambiguity in a message as a stochastic modification of its true value.^5^ Specifically, agent i sees the opinion of agent j as a sample drawn from a Gaussian distribution centred around the opinion $x_{j}$ and truncated at the bounds of the opinion space. The variance of this noise distribution, ν , determines the typical deviation, $\xi_{am}$ , of a sender’s message from its true opinion,


(3.4) $$\begin{array}{l} x_{i}\mapsto \left\{ \begin{array}{l} x_{i}+\mu \cdot (m_{j}-x_{i})\;\text{if }\left|x_{i}-m_{j}\right|\leq \epsilon \\ x_{i}\;\;\;\;else \end{array}\right.\\ \;with\;the\;message\;m_{j}=x_{j}\;+\;\xi_{am}\;where\;\xi_{am}∼N\left(\mu =0,\;\sigma =v\right)\;s.t.\;m_{j}\in \left[0,1\right] \end{array}.$$

When agent i receives the noisy message, $m_{j}$ , it is unaware of the actual value of $x_{j}$ but nevertheless applies the update rule using the noisy message as the best available representation of $x_{j}$ . When noise is negligible, ν→0 , agents see deterministic and accurate opinions as in equation (2.1). Note that owing to the truncation of messages, an agent at the boundary with opinion $x_{j}=1$ , for example, can only send messages with $m_{j}\leq 1$ . That is, in this extreme case, messages $m_{j}$ are drawn from a half-normal distribution with an average message of $1-\sqrt{2/\pi }\cdot \nu$ instead of $x_{j}$ .

Ambiguity noise affects opinion formation directly and indirectly. Through its direct effect, ambiguity noise can temporarily drive agents apart even if their opinions are identical (similar to exogenous or adaptation noise) because the agents are unaware of their actual agreement. Through its indirect effect, ambiguity noise influences whether agents can successfully interact with each other or not (similar to selectivity noise). In particular, when ambiguity noise is strong, ν≫0 , even agents with very distant opinions may occasionally be positively influenced by each other’s opinion when a message deviates so strongly from the sender’s opinion that it creates the illusion for the receiver that the sender’s true opinion is similar to its own. Ambiguity noise affects the message and, thereby, indirectly also the selection and adaptation of a receiver. In contrast, selectivity or adaptation noises affect the receiver directly and independently of each other. Ambiguity noise thus has a different real-world meaning from these previously studied types of noise and leads to non-trivial results that cannot be inferred by simply adding up selectivity and adaptation noise. We, thus, treat ambiguity as an independent source of noise.

### 3.3. Simulation experiments

We are interested in how ambiguity noise affects the emergence of agreement or disagreement in a virtual society and how the results compare with the other types of noise. As a metric for societal disagreement, we use the dispersion of the agents’ opinions (refer to [65] for an overview of measures of disagreement). Additionally, we also consider the kurtosis of the opinion distribution in electronic supplementary material, figures S1 and S2, with similar qualitative results. The dispersion σ is calculated as the standard deviation of the opinion distribution, $\sigma \left(\left\{x_{i}|i\right\}\right)$ , for all agents i at a specific time. A small dispersion indicates that the agents largely agree and the opinion distribution has a narrow (approximately unimodal) shape. A large dispersion indicates disagreement, which can reflect either (i) a polarized society with agents separating into multiple, but internally narrow opinion clusters or (ii) a diffused society with agents forming a single opinion cluster that is dispersed and incohesive.

We perform experiments by fixing the number of agents, n=100 , and the maximum speed of convergence, μ=0.5 , and by varying the level of confirmation bias, which is inversely related to the confidence bound, and the level of noise. For simplicity, we assume that all agents share the same level of bias and noise. Noise is a feature of human behavior that is independent of bias, and, thus, we present our results in terms of the corresponding parameters ν and ϵ . One time step in the model consists of two agents being randomly paired (without network structure or systematic selection preferences) and potentially adapting their opinions. We terminate the simulation after $t=10^{5}$ time steps, that is when a single agent has engaged, on average, in 2000 one-on-one interactions. In the first part of this study, we analyse hypothetical scenarios in which the agents have uniformly distributed initial opinions with a typical initial dispersion σ(t=0)=0.29±0.01 . In the second part, we apply our model to opinion formation in the climate change debate, where opinions represent the concerns of agents about climate change and initial opinions are sampled from a distribution calibrated to survey data [43].

## 4. Results

### 4.1. Ambiguity noise promotes agreement

**Figure 2.**
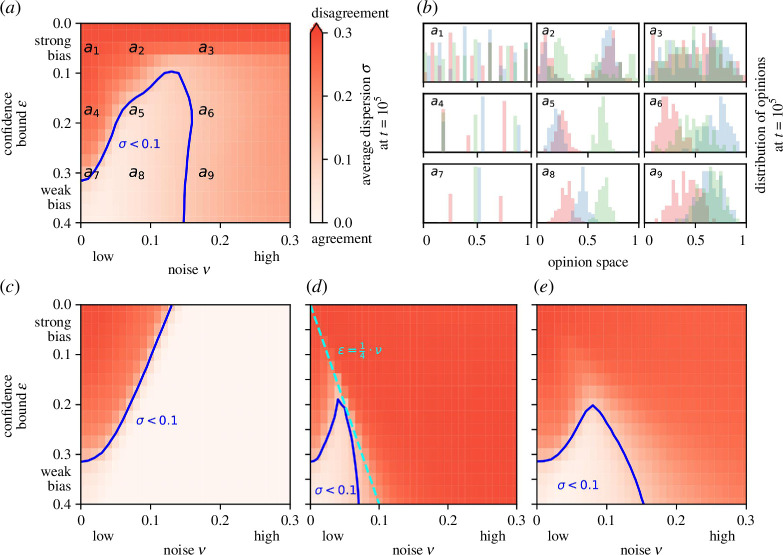
Dispersion, σ , averaged over 1000 stochastic simulations with $t=10^{5}$ steps, under different levels of confirmation bias as a function of the confidence bound ϵ (*y*-axis) and noise ν (*x*-axis), including ambiguity noise $\xi_{am}$ (*a*), selectivity noise $\xi_{se}$ (*c*), adaptation noise $\xi_{ad}$ (*d*) or exogenous noise $\xi_{ex}$ (*e*). In each simulation, the initial opinions of the agents are drawn from a uniform distribution. Low dispersion (light red) indicates strong agreement and high dispersion (deep red) indicates disagreement. For comparison between the panels, the blue line denotes the noise-to-bias ratios for which, on average, σ<0.1 . The cyan line in (*d*) denotes the theoretical transition from agreement to disagreement under adaptation noise derived by Zhang & Zhao [49]. The subpanels in (*b*), corresponding to locations $a_{1}$ to $a_{9}$ in (*a*), show the distributions of the agents’ opinions at $t=10^{5}$ over the opinion space for three exemplary simulations (coloured histograms in *b*) under different combinations of confirmation bias ( ϵ=0.05 , 0.175 or 0.3 ) and ambiguity noise ( $\nu =10^{-10}$ , 0.08 or 0.18 ).

Depending on the level of confirmation bias and noise, the opinions of the agents either converge (low dispersion) or remain in disagreement (high dispersion). When noise is negligible, ν→0 (locations $a_{1}$ , $a_{4}$ , $a_{7}$ in figure 2*a* and the corresponding subpanels in figure 2*b*), agreement emerges only when bias is sufficiently weak, that is when the confidence bound ϵ≳0.3 (location $a_{7}$ ). The number of clusters in the BC model without noise is typically around 1/(2ϵ) . For a moderate bias, ϵ∈[0.1,0.3] , ambiguity noise induces agreement, with dispersion σ→0 (location $a_{5}$ ). This reverses when noise is very high (location $a_{6}$ ). A stronger bias in this regime creates more disagreement but can be compensated for by stronger noise. When the bias exceeds a threshold, here ϵ≲0.075 , ambiguity noise is not sufficient to create agreement within the simulation time considered and the distribution of opinions remains diffuse with σ≈0.3 (locations $a_{2}$ and $a_{3}$ ). This pattern is qualitatively robust for a wide range of parameter choices such as a smaller/larger number of agents n , a shorter/longer simulation time t or a slower speed of convergence μ (see electronic supplementary material, figures S3–S8). The range of bias levels within which noise induces agreement among agents widens with an increasing number of agents n or simulation time t . As such, the dispersion is somewhat sensitive to the model parameters in the critical configuration $a_{5}$ (see electronic supplementary material, figures S4, S6 and S8), but this does not affect the robustness of our main results. In particular, the existence of a transition between disagreement and agreement for moderate ambiguity noise and moderate bias is independent of the parameters reflecting system size, simulation time and convergence speed.

### 4.2. Ambiguity noise induces group drift

Even when agents are in full agreement about their opinions, the mean opinion of the society, $\bar{x}$ , can still change owing to the ambiguity noise in communicated opinions. Opinion distributions can thus become multi-modal even after convergence, and clusters of shared opinions can, theoretically, re-emerge. When bias is moderate, the agents even develop more extreme opinions (figure 2*b*, $a_{5}$ and $a_{6}$ ), that is, the average opinion tends to drift more towards the edges of the opinion space, compared to when bias is weak ( $a_{7}$ – $a_{9}$ ). Electronic supplementary material, figure S9 shows that this drift is a robust feature of the model under moderate ambiguity noise and moderate bias. Drift can be explained by the balance of two opposing forces in the presence of ambiguity noise: repulsion from the bounds of the opinion space and attraction towards extremists. First, agents with extreme opinions are dragged away from the bounds of the opinion space. This is because the opinions of extreme agents can only be pulled in one direction: towards more moderate values. This happens in all variations of the BC model, independent of the type and degree of noise. Second, and acting as a counterforce to the effect of extremists becoming more moderate, agents with extreme opinions in a given distribution tend to be more successful in pulling agents with moderate opinions closer to the bounds of the opinion space. Messages from agents with extreme opinions tend to be seen as more moderate (owing to the boundedness of the opinion space and the truncation of the noise distribution when drawing a ‘noisy message’) and, as a consequence, they are more likely to be accepted by moderate agents. In other words, the agents with relatively extreme opinions benefit from ambiguity, making them more successful in transmitting their messages to receivers than the agents with relatively moderate opinions (see electronic supplementary material, figure S10). As a consequence, the interplay of a moderate confirmation bias with moderate to high ambiguity noise can pull initially diverse and moderate societies towards more extreme average opinions (see also electronic supplementary material, figure S11, which shows how the mean opinion of a Gaussian opinion distribution evolves for different levels of ambiguity noise depending on how extreme the initial opinions are). However, the drift dynamics are non-trivially dependent on the distribution of opinions and drift is a finite-size effect that disappears for larger societies, for example, with *n* = 1000 fully connected agents (electronic supplementary material, figure S3*b*).

### 4.3. Different types of noise induce different opinion patterns

There are some similarities between the results obtained with ambiguity noise (figure 2*a*) and the other three types of noise—selectivity (figure 2*c*), adaptation (figure 2*d*) and exogenous noise (figure 2*e*). In particular, noise can induce agreement for a range of bias levels and, within this range, more bias always requires higher noise to achieve agreement. This result depends on the type of noise considered, especially under relatively strong bias, ϵ≲0.2 . Selectivity noise (figure 2*c*) induces agreement over the full range of bias levels as long as noise is sufficiently high. However, increasing the level of adaptation and exogenous noise (figure 2*d*,*e*) beyond some threshold inhibits the emergence of agreement, causing an abrupt transition from a narrow opinion distribution to a broader one. Thus, reaching an agreement under a strong bias is inhibited regardless of noise. For example, for adaptation noise, agreements break when ν>0.25⋅ϵ (which was also analytically derived in [49]). That is, with a stronger bias (smaller ϵ ), this transition is triggered at lower levels of noise. Agreement emerges only within a narrow range of low adaptation noise, and this range shrinks as bias increases. In comparison with these three types of noise, ambiguity noise generates a different pattern (figure 2*a*): agreement emerges only within a range of moderate noise levels (similar to adaptation and exogenous noise), but this range does not shrink as bias increases somewhat (in contrast to adaptation and exogenous noise and more similar to selectivity noise) such that even a relatively strong bias, 0.2≳ϵ≳0.1 , can be counteracted by ambiguity noise.

### 4.4. Bias and ambiguity noise foster pro-environmental agreement

**Figure 3.**
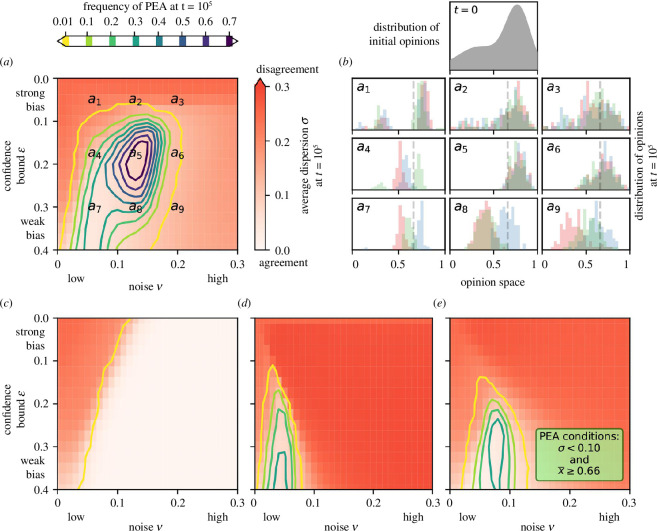
Frequencies of a ‘pro-environmental agreement’ (PEA) obtained from an ensemble of 1000 simulations initialized with a distribution of opinions reflecting the ‘six Americas’ [43] (subpanel t=0 in *b*) for different levels of noise and confirmation bias. PEA is a state of the model defined by low dispersion, σ<0.1 , and high average concern about climate change, $\bar{x}\geq 4/6$ , at time $t=10^{5}$ . Panel (*a*) shows the results for ambiguity noise (as in figure 2) and, correspondingly, subpanels $a_{1}$ – $a_{9}$ in (*b*) show example distributions of agent opinions at $t=10^{5}$ for locations in (*a*). PEA is rarely reached when bias is strong (locations $a_{1}$ – $a_{3}$ ), noise is low ( $a_{4}$ ) or noise is very high ( $a_{3}$ , $a_{6}$ and $a_{9}$ ). PEA is reached in less than 36% of the simulations when bias is weak ( $a_{7}$ – $a_{9}$ ). However, PEA is frequently reached (in 77% of the simulations) when both the bias and the ambiguity noise are moderate, ϵ≈0.175 and ν≈0.13 (location $a_{5}$ ). Under selectivity noise (*c*), adaptation noise (*d*) or exogenous noise (*e*), the maximum frequency of reaching PEA does not exceed 36%, regardless of the noise-to-bias ratio.

For a realistic representation of opinions on climate change in society, we calibrate the initial opinions of the agents to empirical data of Maibach *et al*. [43] (see appendix A for details). A high value $x_{i}$ reflects a strong concern about climate change (and vice versa). Following the six distinct categories obtained in the survey by Maibach *et al*., we define agents as ‘concerned’ if $x_{i}>4/6$ and ‘alarmed’ if $x_{i}>5/6$ . The initial distribution (see subpanel t=0 in figure 3*b*) is skewed towards high concerns, but there is also a significant fraction of dismissive or neutral agents. In the context of climate change, we are interested in the ability of a society to reach a pro-environmental agreement (PEA). We define this PEA as a state in which (i) the agents are, on average, at least concerned (if not alarmed) about climate change, $\bar{x}\geq 4/6$ , and (ii) the agents largely agree on this level of concern, σ≤0.1 . At t=0 , both requirements for a PEA are typically not fulfilled with $\bar{x}(t=0)=0.60\;(\pm 0.03)<4/6$ and σ(t=0)=0.25(±0.01)>0.1 .

**Figure 4.**
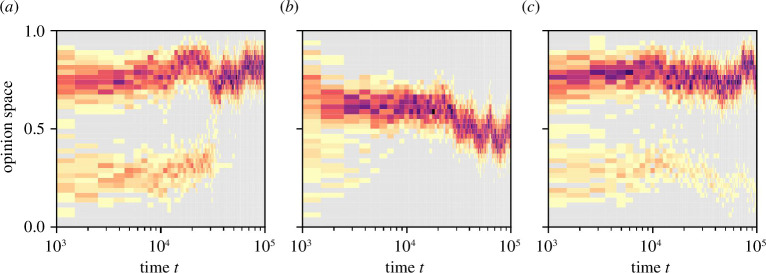
Typical opinion dynamics in simulations with calibrated initial conditions (figure 3) under moderate bias, ϵ=0.175 , and moderate ambiguity noise, ν=0.08 . This configuration lies in the critical region between $a_{4}$ , where PEA is barely reached, and $a_{5}$ , where PEA is very frequently reached. The colour denotes the agent density in each bin over time (note the logarithmic scale on the time axis). Dark red colours represent high density (i.e. many agents have opinions $x_{i}$ falling in the corresponding bin at time t ), bright yellow colours represent low density, and grey represents zero density. A consensus with very low dispersion can be reached relatively early (*a*), late (*b*) or never (within the simulated time horizon) (*c*). In the last case, some agents cluster in the lower half of the opinion space. The mean opinion at $t=10^{5}$ in the simulations in (*a*) and (*c*) are above the threshold for a PEA, $\bar{x}\geq 0.66$ , for at least the second half of the simulation, but the dispersion for the simulation in (*c*) is above the threshold for PEA, σ=0.1 , throughout the simulation and, therefore, only the simulation in (*a*) represents a PEA.

Figure 3*a* shows the frequency of a society-wide PEA resulting from 1000 independent simulations for different bias and ambiguity noise levels. The requirement for reaching an agreement, σ≤0.1 , prevents the formation of a PEA when (i) bias is strong (see locations $a_{1}$ – $a_{3}$ in figure 3*a* and corresponding subpanels in figure 3*b*), (ii) bias is moderate and ambiguity noise is low (location $a_{4}$ ), and (iii) ambiguity noise is high (locations $a_{3}$ , $a_{6}$ and $a_{9}$ ). The requirement for collectively being at least concerned about climate change, $\bar{x}>4/6$ , prevents the formation of a PEA when bias is weak (locations $a_{7}$ – $a_{9}$ ). In particular, in societies with a weak bias, agents tend to reach an agreement, but this agreement comes at the cost of reduced climate change concerns and, consequently, reduced frequency of PEA. There is an optimal noise-to-bias ratio, ϵ≈0.175 and ν≈0.13 (location $a_{5}$ ), for which the society reaches PEA with a much higher frequency (77% of the simulations). For a better understanding of the model behavior, figure 4 shows how the opinions evolve in three example simulations of societies with moderate bias, ϵ=0.13 , and ambiguity noise, ν=0.08 , which represents a critical point where PEA is reached in some cases (figure 4*a*) but not in others owing to a lack of average concern (figure 4*b*) or a lack of agreement (figure 4*c*). Even in cases with general agreement among the agents, opinions still fluctuate (figure 4*a*,*b*). The average opinion, $\bar{x}$ , however, remains quite extreme and stable, especially when ambiguity noise and bias are similar to the configuration $a_{5}$ in figure 3*a* (see electronic supplementary material, figure S12).

Interestingly, we observe this pattern only with ambiguity noise. In contrast, under selectivity noise (figure 3*c*), adaptation noise (figure 3*d*) or exogenous noise (figure 3*e*), the frequency of PEA remains below 36%, regardless of the levels of noise or bias. For instance, in the case of high selectivity noise, agents nearly always reach an agreement but only by compromising with the sceptical minority, and, thus, the consensus opinion is typically not concerned or alarmed, $\bar{x}<0.6$ . This implies that ambiguity noise in social influence probably promotes a scenario in which agents come to agree on a high level of climate change concern, whereas other types of noise do not have such an effect.

### 4.5. Robustness

In this section, we highlight several critical assumptions that are inherent to the results presented so far and briefly examine how altering these assumptions affects our results. First, in line with most other social-influence models, we assume one-to-one interactions in which a single focal agent assimilates to the view of a single sender. In reality, there are situations in which an individual considers the opinions of many others (*many-to-one* interaction [35]), for example when the individual hears about election polls, or situations in which a single individual expresses an opinion publicly to many others simultaneously (*one-to-many* interaction [66]), for example, when the individual posts statements on a social media platform. We find that the assumed communication regime has a pronounced effect on the opinion formation pattern in the presence of ambiguity noise. Under a many-to-one communication regime, the thresholds for drift and agreement resulting from ambiguity noise are much higher than in the one-to-one case. That is, high ambiguity noise is required to induce agreement, and a combination of strong bias and high ambiguity noise is required to obtain drifting. One-to-many communication, in contrast, lowers the threshold for reaching an agreement and generates high-frequency drift. That is, even low levels of noise induce agreement regardless of bias, but the consensus opinion fluctuates across the opinion space. This represents a society in which all individuals share an opinion but, as a collective, they are highly volatile about this opinion. Exemplary simulation runs with these alternative communication regimes can be found in the electronic supplementary material, figures S13–S16. In sum, while a many-to-one interaction may sometimes make disagreement more robust even under relatively high levels of noise, a one-to-many type of interaction might sometimes amplify the interference of noise.

Second, we assume that all agents are connected and thus able to directly influence each other. This assumption may hold for small groups, but social influence patterns are typically more characteristic of networks in which nodes represent agents and links represent influence channels between them (based on social relations), and several studies have shown the influence of network structure on opinion dynamics [7,67,68]. For example, it is well-known that the degree of transitivity (a defining characteristic of human social networks) can critically alter the dynamics of opinions. A thorough analysis of the effects of different network structures on the impact of the different types of noise would go beyond the scope of this study. Our exploratory analysis, however, confirms that the qualitative patterns obtained with a fully connected network are similar to those obtained with a transitive network, such as the standard small-world network [69], thus attesting to the robustness of the model dynamics to substantively different network topologies (electronic supplementary material, figures S17 and S18).

Third, we assume a zero-mean Gaussian noise distribution—arguably the most plausible assumption one can make about stochastic variation in the real world. However, the shape of the noise distribution can have important implications. For example, Gaussian noise is unbounded and, as such, there is a small but non-zero chance that an agent with opinion $x_{i}=1$ communicates a message $m_{i}=0$ if ν>0 . In electronic supplementary material, figure S19, we compare the simulations presented in figure 3 (in which we assume Gaussian noise and calibrated initial conditions) with simulations in which we assume that noise is instead drawn from a zero-mean, bounded uniform distribution, ξ∈[−ν,ν] , for the four types of noise (refer to [54] for a study using the same noise distribution). The results are nearly identical in most cases, although the optimal combination of ambiguity noise and confirmation bias produces much less PEA when noise is drawn from a bounded uniform distribution. Bounded uniform exogenous noise is especially effective in fostering PEA.

## 5. Discussion

We have presented a taxonomy of noise and provided insights into the surprisingly large differences obtained from implementations of these different types of noise in the BC model. In general, noise can induce agreement among moderately biased agents by smoothing out divisions between agents in different clusters. However, the conditions for this effect to occur depend on the type of noise considered. For example, selectivity noise—acting on the connection between agents—always promotes agreement, whereas adaptation and exogenous noise—acting on the agent after or independent of social influence—promote agreement only within a narrow range of bias and noise levels. These results are consistent with the previous modelling studies considering these types of noise (or similar conceptualizations of them) in different opinion dynamics models (see corresponding references in §3). We have focused our analysis predominantly on ambiguity noise because its effects on consensus formation have been the least extensively addressed in prior models of social influence. By affecting communicated messages rather than their recipients, ambiguity noise has indirect consequences for the two relevant opinion formation processes in our model: selection and adaptation to social influence. It seems intuitive to think that clarity and unbiased reasoning in public debates are key to enabling a society with initially diverse or polarized opinions to reach an agreement [28,70]. However, clear communication might be at odds with successful consensus formation, as indicated by conceptual work on ambiguity as a strategic tool [26,71]. Our results show that moderate ambiguity in expressed opinions facilitates agreement under a wide range of bias levels—a pattern that differs from those of selectivity and adaptation noise.

To go beyond hypothetical scenarios of uniform initial opinions (as assumed in most formal models [14,57]), we initialized our model with a data-driven distribution of opinions about climate change [43]. According to this distribution (and in line with other surveys on climate change opinions [23,72]), the majority of people are somewhat concerned about climate change (with 18% in the ‘alarmed’ and 33% in the ‘concerned’ categories), but people appear far from having reached a consensus (with significant fractions of 19% in the ‘cautious’, 12% in the ‘disengaged’, 11% in the ‘doubtful’ and 7% in the ‘dismissive’ categories in 2008) [43]. In some cases, opinion diversity can be beneficial to collective decision-making, for example, when diversity prevents conformity, groupthink or overconfidence [73,74]. However, as a large body of climate experts judge climate change as a ‘threat to human well-being and planetary health’ [75] and call for an ‘emergency response’ [76], continued disagreement among large sectors of the population may undermine the support for policies aiming to produce an adequate response.

Surprisingly, we found that a group of agents affected by a moderate bias and a medium ambiguity noise provided the best conditions for aligning towards a high level of concern about climate change. The results of the climate change scenario are qualitatively similar to the hypothetical one, but they highlight even more prominently the separate effects of different noise types. With the help of normally distributed ambiguity noise, agents can reach an agreement under a moderate bias, and, crucially, this agreement is reached without having to compromise the majority’s pro-environmental attitudes. In this scenario, ambiguity noise clearly outperforms other types of noise and produces the unique pattern of a society driving itself towards a shared, extreme opinion. Of course, we should be cautious about drawing strong conclusions about predictions or prescriptions concerning climate change opinions, as the way those opinions are formed and updated surely involves many factors not included in our model. Nevertheless, our model provides a good jumping-off point for such considerations.

Mathematical models, even the most sophisticated ones, are obviously only approximations of real-world social processes. For example, our model leaves aside media influence, social structure, value systems, ideology or trust in experts. While we are confident in the robustness of the effects of ambiguity noise, there are several avenues to increase the realism of the model. We focus here on three that we deem particularly promising. First, people have only a few salient social influences [77] with homophily constraining the sources of those influences [78]. Our analysis indicates that embedding agents in a small-world network does not critically alter the qualitative results regardless of the network parameters (see §4.5). Still, future research could investigate how homophily, represented as a bias in the selection of partners in such networks (as in [16,79]), alters the opinion dynamics. Second, ambiguity is not entirely unintended. People strategically adapt the degree of ambiguity when expressing opinions to their peers [26,27,70,80]. By assuming that the noise in the model is heterogeneous and adaptive, future research could investigate how agents develop communication strategies that use ambiguity strategically to spread opinions to their peers more effectively. Similarly, future research could investigate how the assumption that confidence bounds are heterogeneous and adaptive affects our results. Third, we focused on studying each type of noise separately, favouring a comparison of their contrasting effects and neglecting their interplay. Future research could investigate how interactions between the different types of noise affect opinion patterns (as in the study by Grauwin & Jensen [36], which explored the effects of interacting selectivity and exogenous noise).

Social influence is only one of the many factors shaping opinions, but it plays a central role in many political debates [81], and particularly in the debate on climate change [30,31,82]. We have argued here that ambiguity in communicated messages is an inherent feature of such social influence and is particularly pronounced when dealing with a complex topic like climate change. There are ways to reduce ambiguity noise in a social debate, for example, by enforcing more transparent and rigorous communication [25] or by fostering the use of clearly identifiable markers [70], such that socially transmitted opinions are more representative of the actual opinions (e.g. by wearing clothes items or using hashtags associated with the support for a particular opinion [83]). Our study, however, supports literature promoting the benefits of noise or ambiguity in communication to foster agreement across society (e.g. [26,28,84–87]. Our findings imply that in the presence of confirmation bias, which is a crucial cognitive factor in the climate change debate [41,42], communication that leaves some space for ambiguity might prove beneficial not only for reaching an agreement but also for strengthening a shared concern on a topic like climate change. This is an important preliminary step for effective policy-making to address such political challenges, but one should be aware of the different kinds of noise and their different impacts on collective opinion formation.

## Data Availability

The model is coded in python [Python Software Foundation. Python Language Reference, version 3.9.5. Available at http://www.python.org]. To foster reproducibility, transparency, and flow of ideas, we make the code publicly available at github.com/PeterSteiglechner/noise-in-OD and have archived it within the Zenodo repository [87]. We have also implemented the model in the defSim package [88] to facilitate investigations on how these types of noise affect opinion patterns under different model assumptions. Electronic supplementary material is available online at [89].

## Footnotes

Though refer to Gigerenzer *et al*. [4] for a perspective on why so-called biases may be adaptive responses to uncertainty.

Other researchers have modelled confirmation bias in the context of collective problem solving using quite different modelling frameworks, but have nevertheless operationalized it similarly as a preference for solutions close to the individual’s current solution [44,45].

The temperature analogy refers to higher chances of interaction between random particles in hot versus cold systems.

For instance, adaptation noise may lead to the situation where an agent is positively influenced by another agent (because their opinions are within the confidence bound), yet ends up disagreeing more with that agent after their interaction. Assume $x_{j}>x_{i}\;and\;x_{j}-x_{i}<\in$ . Although $x_{i}$ should increase owing to the positive social influence, it may decrease if $\xi_{ad}\ll 0$ . For example, when two individuals already hold very similar opinions, a discussion could lead them to further converge. But, if this conversation prompts them to reconsider certain arguments differently, they might end up disagreeing more than they did before their interaction.

We acknowledge that the term ambiguity is in itself ambiguous. Ambiguity might imply that recipients see multiple coherent interpretations of a socially transmitted message. Here, we define ambiguity noise in the following sense. Although agents receive a message as a single value, this value is coherent with multiple possible inferences of the true opinion of the sender. For readers objecting to our use of ambiguity, we propose substituting ‘ambiguity noise’ with ‘message noise’ or ‘transmission noise’.

## Appendix A. Calibrating initial opinions on climate change to empirical data

To apply the model to the climate change debate, we use empirical data from Maibach *et al*. [43] to determine the distribution of the initial opinions. In 2008, the authors surveyed more than 2000 US citizens about their global warming beliefs, environmental behaviours, policy preferences and issue engagement. They identified six distinct categories of attitudes towards climate change in the survey population: dismissive (7% of the sampled population), doubtful (11%), disengaged (12%), cautious (18%), concerned (33%) and alarmed (19%). To reflect this distribution of opinions at the beginning of our simulations, we divide the continuous opinion space, x∈[0,1] , into six equally sized segments, where each segment represents the opinion range of one of the six categories. For example, agents with x∈[5/6,1] are ‘alarmed’ and agents with x∈[4/6,5/6] are ‘concerned’. We draw initial opinions from a smooth distribution, which we fitted to match the population shares from Maibach *et al*. [43] within each segment (using a superposition of two Gaussian functions, see subpanel t=0 in figure 3*b*).
